# *Lactobacillus johnsonii* CCFM1376 improves hypercholesterolemia in mice by regulating the composition of bile acids

**DOI:** 10.20517/mrr.2024.38

**Published:** 2024-11-09

**Authors:** Kexue Chen, Danting Dang, Huizhen Li, R. Paul Ross, Catherine Stanton, Wei Chen, Bo Yang

**Affiliations:** ^1^State Key Laboratory of Food Science and Resources, Jiangnan University, Wuxi 214122, Jiangsu, China.; ^2^School of Food Science and Technology, Jiangnan University, Wuxi 214122, Jiangsu, China.; ^3^International Joint Research Laboratory for Maternal-Infant Microbiota and Health, Jiangnan University, Wuxi 214122, Jiangsu, China.; ^4^APC Microbiome Ireland, University College Cork, Cork T12 X4H4, Ireland.; ^5^Teagasc Food Research Centre, Moorepark, Cork P61 C996, Ireland.; ^6^National Engineering Research Center for Functional Food, Jiangnan University, Wuxi 214122, Jiangsu, China.

**Keywords:** *Lactobacillus johnsonii*, bile salt hydrolase, bile acids, FXR

## Abstract

**Aim:** Strains with high bile salt hydrolase (BSH) activity have the potential to regulate cholesterol metabolism. This study aimed to assess the alleviating effect of *Lactobacillus johnsonii* (*L. johnsonii*) CCFM1376, a strain with high BSH activity, on mice with hypercholesterolemia and explore the mechanism of its effect through the modulation of bile acid metabolism.

**Methods:** The BSH activity was measured using the ninhydrin method. C57BL/6J mice were given a high-cholesterol diet to induce hypercholesterolemia with simultaneous gavage of *L. johnsonii* CCFM1376 for 8 weeks. The biochemical parameters in the serum and liver of hypercholesterolemic mice were measured to assess the alleviating effect of *L. johnsonii* CCFM1376 on hypercholesterolemia. Bile acid content in the mouse liver, serum, distal ileum contents, and feces was determined using liquid chromatograph mass spectrometer (LC-MS). RNA was extracted from mouse ileum and liver, and the expression levels of relative genes implicated in bile acid metabolism were measured by quantitative real-time PCR (qPCR).

**Results:** Compared to the model group, the group treated with *L. johnsonii* CCFM1376 exhibited significantly reduced levels of serum total cholesterol (TC) and low-density lipoprotein cholesterol (LDL-C), along with a significant increase in high density lipoproteins cholesterol (HDL-C) level. Moreover, hepatic levels of TC and LDL-C in the CCFM1376 group also decreased significantly. Furthermore, the content and amount of unconjugated bile acids in the hepatic-enteric circulation of the *L. johnsonii* CCFM1376 group significantly increased, and the total bile acid content in the feces also significantly increased. In the *L. johnsonii* CCFM1376 group, the relative expression levels of ileal farnesoid X receptor (FXR) and fibroblast growth factor 15 (FGF15) were downregulated, while the relative expression level of CYP7A1 was upregulated.

**Conclusion:** These results indicated *L. johnsonii* CCFM1376 improves hypercholesterolemia in mice by regulating the composition of bile acids. This provides a reference for probiotic strategy to regulate cholesterol metabolism.

## INTRODUCTION

It is widely acknowledged that disorders impacting the cardiovascular and cerebrovascular systems are significant in terms of global death rates, resulting in millions of fatalities each year. With economic development and improved living conditions, dietary habits have gradually shifted toward high-fat, high-cholesterol, and high-calorie foods, leading to a substantial rise in the number of people with dyslipidemia^[1]^. Hypercholesterolemia is distinguished by increased amounts of cholesterol in the blood plasma, particularly low-density lipoprotein cholesterol (LDL-C), and epidemiological studies consistently indicate that this is a major risk factor for a range of cardiovascular diseases, including atherosclerosis^[2]^.

Probiotics with high bile salt hydrolase (BSH) activity can significantly reduce serum cholesterol levels in humans and animals^[3]^. High BSH activity is also a key criterion for screening probiotics with cholesterol-lowering functions in many current research studies^[4]^. BSH is an enzyme widely present in the gut microbiota of humans and other mammals, facilitating the hydrolysis of conjugated bile acids into unconjugated bile acids and amino acids (glycine or taurine), thereby regulating the metabolism of bile acid. BSH is predominantly produced by microbes such as *Lactobacillus*, *Bifidobacterium*, *Enterococcus*, and *Clostridium* in the intestine. *Lactobacillus* and *Bifidobacterium* are the main sources for the *in vitro* screening of high-activity BSH strains^[5]^.

Hepatocytes in the liver synthesize primary bile acids from cholesterol through two interconnected pathways, the classic and alternative pathways. These bile acids are then conjugated by combining with glycine or taurine^[4]^. When these conjugated bile acids are excreted into the ileum or upper colon, they undergo hydrolysis due to the effect of BSH enzymes generated by intestinal flora, releasing unconjugated bile acids and amino acids. Subsequently, unconjugated bile acids are subject to additional metabolic changes, yielding secondary bile acids, specifically deoxycholic acid (DCA) and lithocholic acid (LCA), under the action of a series of microbial enzymes, such as 7α-dehydroxylase^[6]^. In the terminal part of the small intestine, most conjugated bile acids are ingested by intestinal epithelial cells through sodium-dependent bile acid transport proteins and transported to the portal vein system, while unconjugated bile acids are less readily reabsorbed. These bile acids circulate back to the liver with the blood and are subsequently embraced by hepatocytes through specific transport proteins, such as the polypeptide responsible for sodium and taurocholate cotransport, along with polypeptides that transport organic anions, with some bile acids participating in the synthesis of cholesterol through the de novo synthesis pathway^[7]^. This forms the hepatic-enteric circulation of cholesterol and bile acids in the human body^[8]^. When BSH activity in the gut increases, more conjugated bile acids are altered into unconjugated bile acids, raising the proportion of unconjugated bile acids. Due to the stronger hydrophobicity of unconjugated bile acids, they are less readily ingested by the gut and are discharged together with feces. Consequently, with fewer bile acids available, the liver is stimulated to synthesize more bile acids from cholesterol to maintain the enterohepatic bile acid balance^[8]^. Additionally, the compositional changes in bile acids influenced the farnesoid X receptor (FXR) signaling pathway^[9]^. The FXR, which is activated by bile acids, acts as a transcription factor that functions as a ligand-activated receptor^[10]^. Different bile acids are capable of acting as messenger molecules to activate or inhibit FXR, making the distribution and variation of the bile acid composition a material basis for the impact of BSH on FXR expression. The control of bile acid transport is affected by the expression of genes encoding ileal bile acid-binding proteins and organic solute transport proteins α/β (OSTα/β), which are both substantially expressed in the ileum when FXR is activated^[11]^.

Previous research has found that *Lactobacillus johnsonii* (*L. johnsonii*) commonly possesses a greater number of *BSH* genes^[12]^. However, the BSH activity and its capacity to regulate cholesterol metabolism in *L. johnsonii* are not yet well-defined. *L. johnsonii* CCFM1376 was isolated from a fecal sample of an adult female and identified as *L. johnsonii* by 16S rDNA sequencing results. This study focuses on the high BSH activity strain, *L. johnsonii* CCFM1376, to evaluate its ability to alleviate hypercholesterolemia in mice. Additionally, the study explores the pathways through which it may ameliorate hypercholesterolemia by modulating bile acid metabolism.

## METHODS

### Quantitative determination of BSH activity

The activated bacterial suspension was inoculated into 20 mL of MRS medium at a 1% volume ratio and statically cultured at 37 °C for 24 h. Post-fermentation, the broth underwent a 10-minute centrifugation process at 10,000 × *g* and a temperature of 4 °C, which effectively separated the bacterial cells. The cell pellet was washed twice and recentrifuged and resuspended the pellet in physiological saline. Adjust the bacterial suspension concentration to an optical density (OD) of 1 at a wavelength of 600 nm to standardize the concentration of the bacterial suspensions. In addition, 1 mL sample of the bacterial suspension with adjusted concentration was treated with an ultrasonic disrupter (Ningbo Xinzhi Biotechnology Co., Ltd.) for 3 min (with a duty cycle ratio of 2:3). Post-treatment, the solution underwent a second centrifugation for 10 min at 10,000 × *g* and 4 °C to precipitate the cellular fragments, yielding a clear cell-free extract. Subsequently, 0.1 mL of the supernatant was combined with an equal volume of a 200 mM conjugated bile salt solution and 1.8 mL of a 0.1 M phosphate buffer at pH 6.0, followed by a 30-minute incubation period at 37 °C with constant agitation. After incubation, a portion of 0.5 mL was extracted from the mixture and subsequently combined with an equal volume of a 15% trichloroacetic acid solution to cease the reaction process, followed by thorough mixing and centrifugation at the maximum speed of the centrifuge for 10 min at 4 °C to collect the supernatant. Subsequently, 0.1 mL of the supernatant was combined with 1.9 mL of ninhydrin reagent, stirred well, and heated in boiling water for 15 min. After cooling to ambient temperature, the mixture’s absorbance was then determined at a wavelength of 570 nm. Standard curves were prepared using glycine and taurine, respectively. The total BSH enzyme activity is defined as the amount of substrate, in micromoles per minute per milliliter, that is hydrolyzed by the crude enzyme to produce amino acids from conjugated bile salts per unit volume per unit time, with the unit expressed as μmol (min·mL)^-1^. The calculation formula is: TA = 4/3 Caa, where Caa represents the concentration of amino acids^[13]^.

### Animal experiments

The animal study was approved by the Jiangnan University Experimental Animal Management and Animal Welfare Ethics Committee (JN.No20230415c1000801[117]). After a week-long acclimation, thirty-two 4-week-old male C57BL/6J mice were evenly allocated into four distinct groups: the control group, the model group, the CCFM1376 group, and the QJSWX160M2 group (*n* = 8). Throughout their 8-week rearing phase, the mice in the control and model groups received 200 μL of physiological saline daily through oral gavage, whereas mice in the *L. johnsonii*-supplemented groups were daily given oral gavage of 200 μL of *L. johnsonii* at a concentration of 1 × 10^10^ colony-forming units (CFU)/mL. The control group was given a standard reference feed, while the other three groups were given the high-cholesterol feed. The standard reference feed (TP23302) and a high-cholesterol feed (TP28600), characterized by 15% fat, 1.25% cholesterol, and 0.5% bile salts, were both sourced from Trophic Animal Feed High-Tech Co., Ltd in China^[14]^.

### Determination of biochemical parameters in mouse serum and liver

Biochemical parameters in mice serum, such as total cholesterol (TC), triglyceride (TG), LDL-C, and high density lipoproteins cholesterol (HDL-C), were assessed using a Mindray fully automated biochemical analyzer (Shenzhen Mindray Animal Medical Technology Co., LTD). The levels of TC, TG, and LDL-C in mouse liver were determined according to the instructions of the assay kits provided by Nanjing Jiancheng Bioengineering Institute, Nanjing, China^[15]^.

### RNA extraction and gene expression analysis in tissues

For 50 mg liver or ileum tissue samples, TRIzol reagent, 500 μL in volume, was introduced to the mixture, which was then thoroughly homogenized using a high-throughput grinder at 4 °C to fully disrupt the cells. Subsequently, the mixture was treated with 200 μL of DNase- and RNase-free water, followed by centrifugation at 12,000 × *g* and 4 °C for 15 min to retrieve the supernatant. The supernatant was mixed with an equal volume of isopropanol, followed by centrifugation at 12,000 × *g* and 4 °C for 10 min to pellet the precipitate. The supernatant was aspirated off, and the pellet was washed twice with 75% ethanol and air-dried. The pellet was resuspended in DNase- and RNase-free water, and complementary DNA (cDNA) was prepared through a reverse transcription reaction according to the instructions of the reverse transcription kit (R333-01 Vazyme Biotech Co., Ltd., Nanjing, China). Gene expression levels within the mouse liver and ileum tissues were quantified through quantitative real-time polymerase chain reaction, conducted on a BioRad thermocycler with the quantitative real-time PCR (qPCR) Master Mix from Vazyme Biotech Co., Ltd, Nanjing, China^[16]^.

### Determination of bile acid content in tissue and feces

Twenty mg freeze-dried feces, 20 mg ileal contents, and 20 mg liver were weighed into a 1.5 mL centrifuge tube. One mL of chromatography-grade methanol was added for homogenization; 50 μL of serum was added into a 1.5 mL centrifuge tube, and 1 mL of chromatography-grade acetonitrile was added for homogenization. After incubation at room temperature for an hour, the sample was centrifuged under cold conditions (4 °C, 12,000 × *g*, 15 min) to isolate the supernatant for subsequent use. The pellet was then treated with 1 mL of methanol, thoroughly mixed, incubated for 15 min, and centrifuged again under the same cold conditions. The supernatants were pooled, and the process was reiterated. Subsequently, the pooled supernatant was freeze-dried to remove all liquid content, followed by the addition of 1 mL of chromatographic-grade methanol for dissolution, and then centrifuged once more under the same conditions. The supernatant was filtered through a 0.22-micron filter, and an aliquot of the sample was transferred into an injection vial for analysis.

For the determination of bile acid content, liquid chromatograph mass spectrometer (LC-MS) was employed with a Thermo U3000 system equipped with an ACQUITY UPLC® HSS T3 1.8 µm column (2.1 × 100 mm). The auto-sampler was maintained at 15 °C, with a flow rate set to 0.30 mL/min, and the column temperature regulated at 35 °C. An injection volume of 2 µL was utilized for the gradient elution, which involved a mobile phase consisting of 1 mM ammonium acetate in water (A) and in methanol (B). The gradient elution program is as follows: 0 min, 20% B; 0-6 min, 20%-60% B; 6-26 min, 100% B; 26-28 min, 100%-50% B; 28-30 min, 50%-20% B; 30-32 min, 20% B^[17]^.

### Statistical analyses

The results of the data are displayed as means ± standard deviations. SPSS software was employed for analysis of variance (ANOVA), with Tukey’s test for subsequent inter-group comparisons. If the *P*-value from the test for variance homogeneity is less than 0.05, a non-parametric alternative is considered^[18]^. In graphical representations, the lack of identical letters between groups denotes a significant discrepancy (*P* < 0.05).

## RESULTS

### BSH activity of *L. johnsonii* CCFM1376

**Figure 1:**
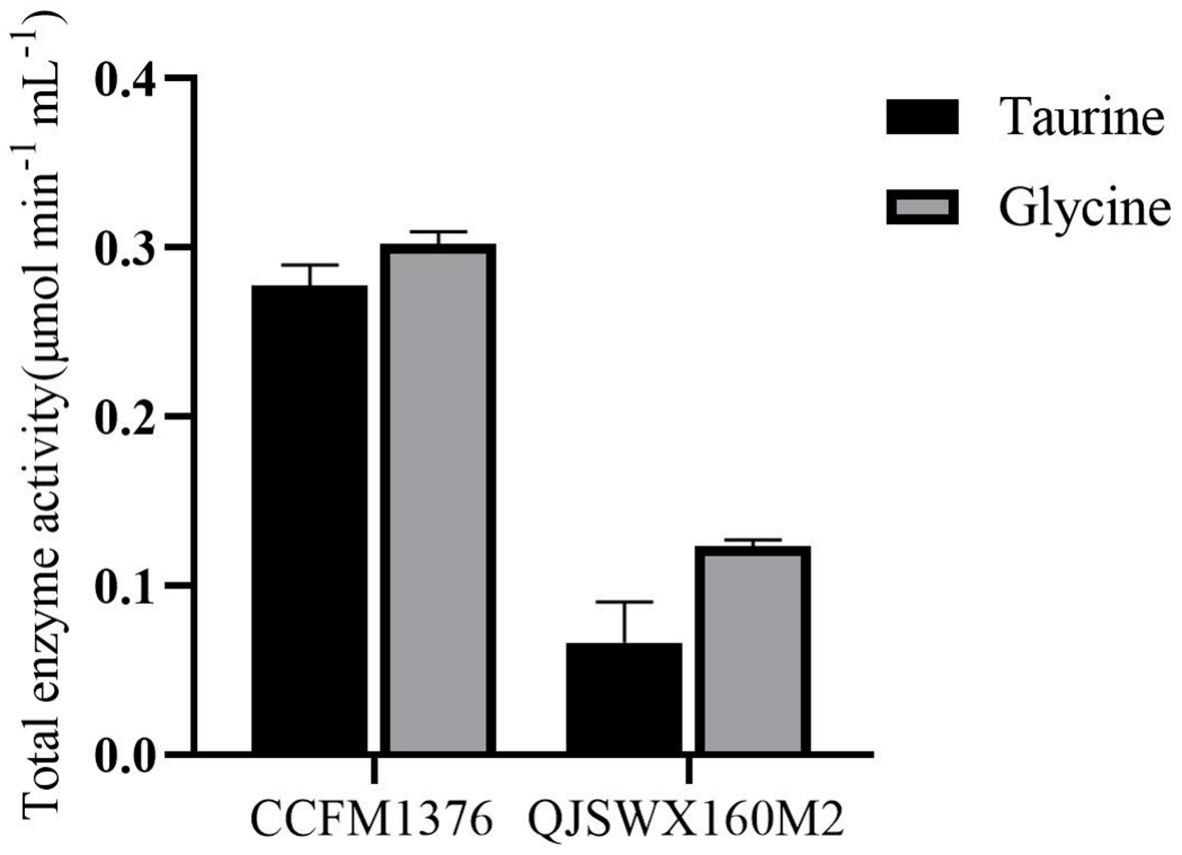
The BSH activity of *L. johnsonii* CCFM1376 and *L. johnsonii* QJSWX160M2. BSH: Bile salt hydrolase.

BSH can catalyze the hydrolysis of conjugated bile acids to form unconjugated bile acid bile acids and amino acids (taurine and glycine) during the bile acid synthesis process. Strains with high BSH activity can promote the enterohepatic circulation of cholesterol and bile acids in the body. Therefore, BSH activity is one of the key factors in assessing a strain’s ability to regulate cholesterol metabolism. This study measured the BSH activity of *L. johnsonii* CCFM1376 and *L. johnsonii* QJSWX160M2 to assess their potential to alleviate hypercholesterolemia. *L. johnsonii* CCFM1376 exhibits hydrolytic activities of 0.2780 μmol·min^-1^·mL^-1^ for taurodeoxycholic acid (TDCA) and 0.3022 μmol·min^-1^·mL^-1^ for glycodeoxycholic acid (GDCA), which are significantly higher than those of *L. johnsonii* QJSWX160M2, with activities of 0.0664 μmol·min^-1^·mL^-1^ for TDCA and 0.1237 μmol·min^-1^·mL^-1^ for GDCA [Figure 1].

### The impact of *L. johnsonii* CCFM1376 on serum lipid levels in hypercholesterolemic mice

**Figure 2:**
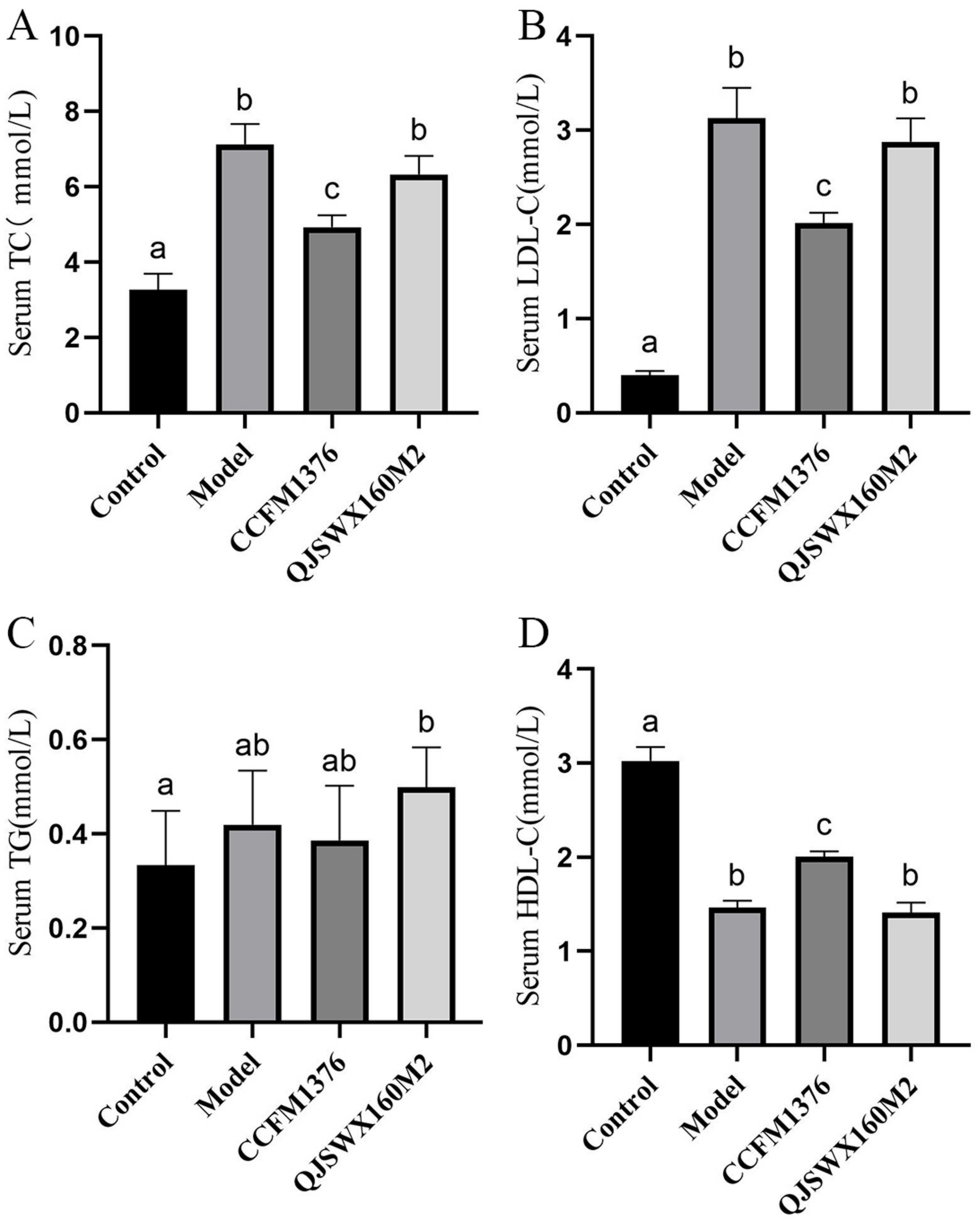
The effect of *L. johnsonii* CCFM1376 on the serum biochemical parameters of hypercholesterolemic mice. (A) TC; (B) LDL-C; (C) TG; (D) HDL-C. Groups with the same lowercase letter did not exhibit significant differences, whereas those with different letters indicated significant differences (*P* < 0.05). TC: Total cholesterol; LDL-C: low-density lipoprotein cholesterol; TG: triglyceride; HDL-C: high density lipoproteins cholesterol.

The main characteristic of hypercholesterolemia is an excessive increase in plasma cholesterol, especially the “bad” cholesterol known as LDL-C. Epidemiological studies consistently indicate that this contributes significantly to the onset of atherosclerosis and a range of cardiovascular diseases. To explore the impact of *L. johnsonii* CCFM1376 on the serum lipid levels in mice with hypercholesterolemia, this study measured the levels of TC, LDL-C, HDL-C, and TG in mouse serum, with a particular focus on TC and LDL-C as key indicators for assessing hypercholesterolemia. After 8 weeks of a high-cholesterol diet, the levels of serum TC and LDL-C in the mice of the model group were significantly higher compared to those in the control group mice [*P* < 0.05, Figure 2A and B]. Compared to the model group, mice supplemented with *L. johnsonii* CCFM1376 demonstrated a considerable drop in the concentration of serum TC and LDL-C (*P* < 0.05). However, *L. johnsonii* QJSWX160M2 did not have these effects. Meanwhile, no considerable changes were noted in TG levels observed across all groups [*P* < 0.05, Figure 2C]. Moreover, mice in the *L. johnsonii* CCFM1376 group demonstrated significantly higher levels of HDL-C in comparison with the model group [*P* < 0.05, Figure 2D].

### The impact of *L. johnsonii* CCFM1376 on hepatic lipid deposition in hypercholesterolemic mice

**Figure 3:**
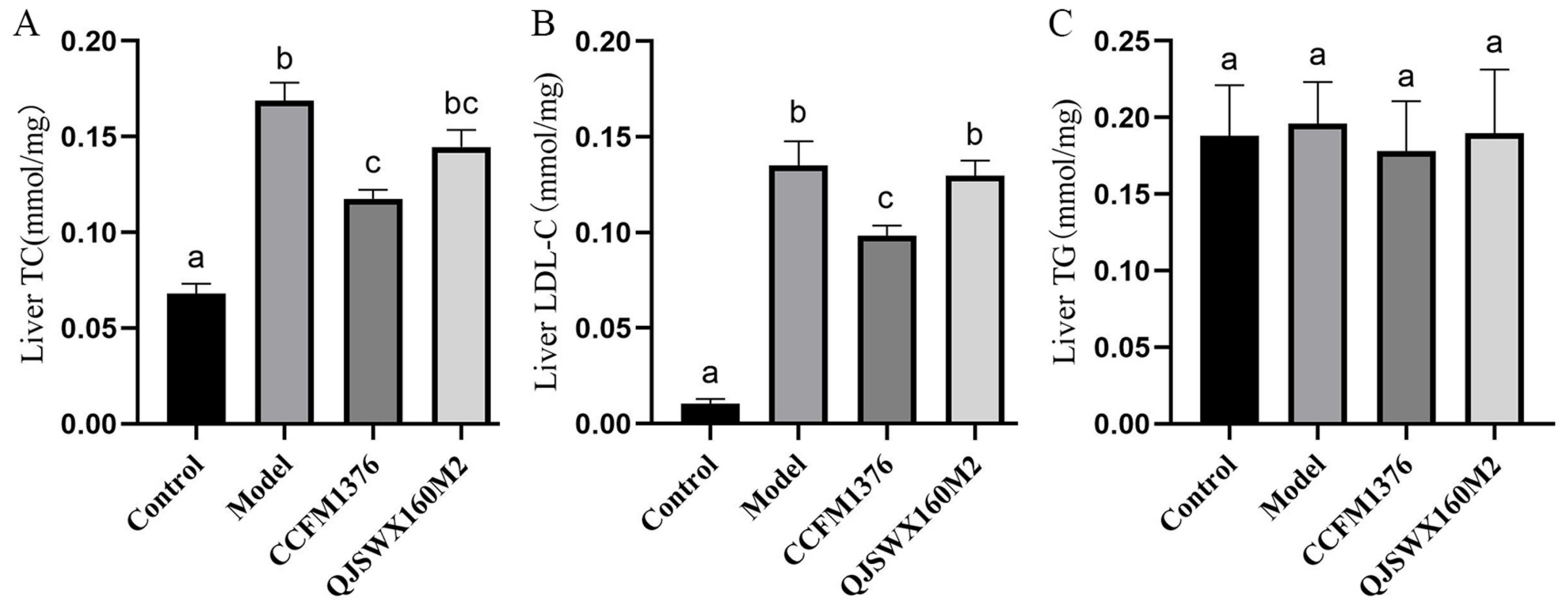
The effect of *L. johnsonii* CCFM1376 on the liver biochemical parameters of hypercholesterolemic mice. (A) TC; (B) LDL-C; (C) TG. Groups with the same lowercase letter did not exhibit significant differences, whereas those with different letters indicated significant differences (*P* < 0.05). TC: Total cholesterol; LDL-C: low-density lipoprotein cholesterol; TG: triglyceride.

**Figure 4:**
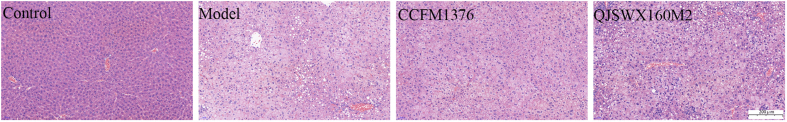
Histopathological analysis of *L. johnsonii* on liver tissue in hypercholesterolemic mice. H&E staining of the liver, 200×. H&E: Hematoxylin-eosin.

To determine whether the supplementation of *L. johnsonii* CCFM1376 can alleviate liver injury caused by a high-cholesterol diet, the levels of TC, TG, and LDL-C in the liver were assessed. In contrast with the control group, the model group exhibited notably higher levels of liver TC and LDL-C [*P* < 0.05, Figure 3A and B]. However, the supplementation with *L. johnsonii* CCFM1376 significantly reduced the levels of TC and LDL-C in the livers of mice with hypercholesterolemia. There were no significant differences in liver TG levels among the groups [*P* < 0.05, Figure 3C]. The application of hematoxylin-eosin (H&E) dye to liver slices showed that in comparison with the control group, the mice in the model group showed more central venous and portal venous congestion in the central areas of the hepatic lobules, surrounded by hepatocytes and sinusoids arranged in an approximate radiating pattern [Figure 4]. The model group showed a higher incidence of hepatocellular fatty degeneration, with a small number of variably sized round vacuoles visible within the cytoplasm. There was significant hepatocellular ballooning degeneration, characterized by a balloon-like swelling of hepatocytes, with the nucleus being centrally located or displaced to one side, and the cytoplasm exhibiting a sparse or fine reticular structure. The group treated with *L. johnsonii* CCFM1376 demonstrated a decline in hepatocellular fatty degeneration and a decline in the round vacuoles within the cytoplasm.

### *L. johnsonii* CCFM1376 alters the hepatic and intestinal bile acid composition in hypercholesterolemic mice

**Figure 5:**
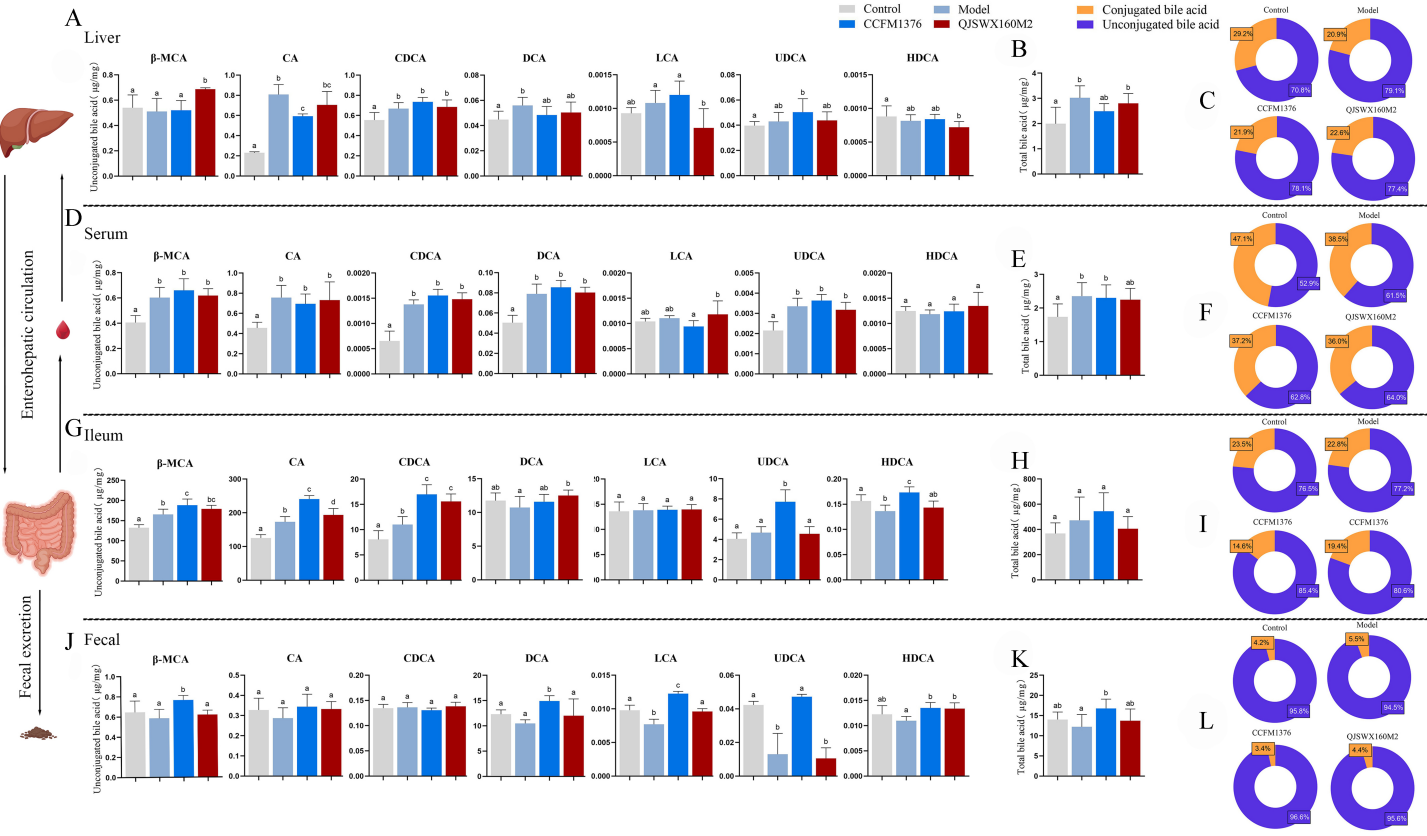
The effect of *L. johnsonii* CCFM1376 on the bile acid composition in the enterohepatic circulation of hypercholesterolemic mice. (A) Unconjugated bile acid composition in the liver; (B) Total bile acid in the liver; (C) bile acid proportion in the liver; (D) unconjugated bile acid composition in the serum; (E) Total bile acid in the serum; (F) bile acid proportion in the serum; (G) unconjugated bile acid composition in the ileum; (H) Total bile acid in the ileum; (I) bile acid proportion in the ileum; (J) unconjugated bile acid composition in the feces; (K) Total bile acid in the feces; (L) bile acid proportion in the feces. Groups with the same lowercase letter did not exhibit significant differences, whereas those with different letters indicated significant differences (*P* < 0.05).

**Figure 6:**
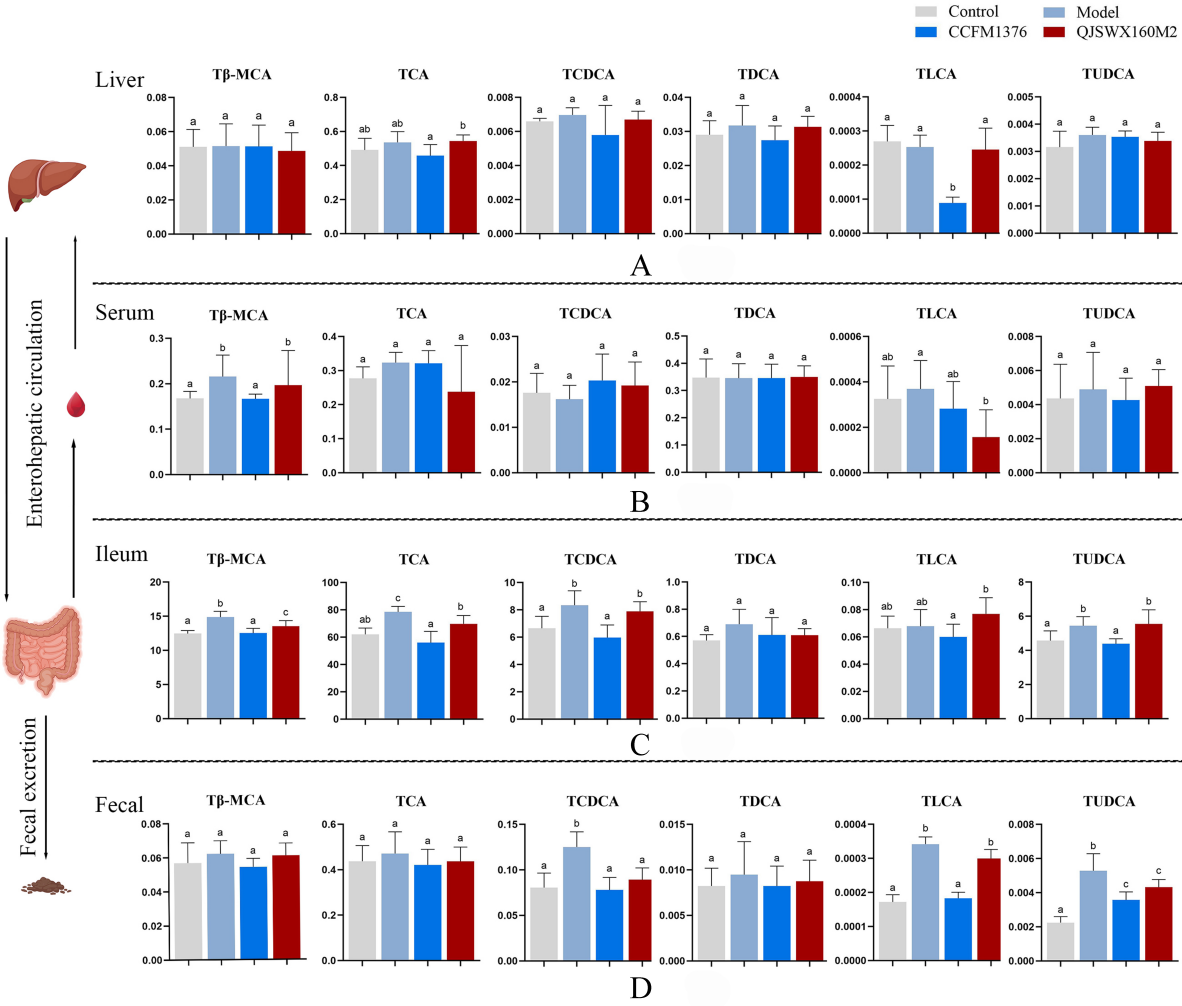
The effect of *L. johnsonii* CCFM1376 on the conjugated bile acid composition in the enterohepatic circulation of hypercholesterolemic mice. (A) Conjugated bile acid composition in the liver; (B) conjugated bile acid composition in the serum; (C) conjugated bile acid composition in the ileum; (D) conjugated bile acid composition in the feces. Groups with the same lowercase letter did not exhibit significant differences, whereas those with different letters indicated significant differences (*P* < 0.05).

The impact on bile acid composition represents the most direct pathway through which strains with high BSH activity exert their effects on cholesterol metabolism. To ascertain the influence of *L. johnsonii* CCFM1376 on the bile acid content in mice, this study conducted targeted quantitative measurements of bile acids in the liver, serum, distal ileum contents, and feces of the mice. The high-cholesterol diet significantly increased the levels of cholic acid (CA) in the livers of mice [*P* <0.05, Figure 5A]. In comparison with the model group, *L. johnsonii* CCFM1376 significantly reduced the hepatic CA levels in mice with hypercholesterolemia. In the serum, levels of β-muricholic acid (β-MCA), CA, chenodeoxycholic acid (CDCA), LCA, and ursodeoxycholic acid (UDCA) in mice on a high-cholesterol diet were all significantly higher than those in the control group. However, no considerable divergences were noted between the *L. johnsonii* CCFM1376 group and the model group [*P* <0.05, Figure 5B]. The ileum, which is rich in bile acids, showed a significant increase in the levels of several unconjugated bile acids, including β-MCA, CA, CDCA, UDCA, and hyodeoxycholic acid (HDCA), in the *L. johnsonii* CCFM1376 group in comparison with the model group [*P* < 0.05, Figure 5C]. Additionally, the levels of β-MCA, DCA, LCA, UDCA, and HDCA in the feces exhibited a marked rise in the *L. johnsonii* CCFM1376 group than in the model group [*P* < 0.05, Figure 5D]. Compared to the control group’s total bile acid content, the *L. johnsonii* CCFM1376 group only showed a significant increase in total bile acid content in the feces, while there were no significant changes in the total bile acid content of the liver, serum, or ileal contents. [*P* < 0.05, Figure 5E-H]. In the *L. johnsonii* CCFM1376 group, the proportion of unconjugated bile acids in the liver, serum, ileal contents, and feces exhibited varying degrees of change, with a notably increased proportion of unconjugated bile acids in the ileum [Figure 5I-L]. Correspondingly, the levels of conjugated bile acids have also changed [Figure 6].

### *L. johnsonii* CCFM 1376 alters the expression profile of genes involved in the regulation of bile acid synthesis mediated by the FXR pathway

**Figure 7:**
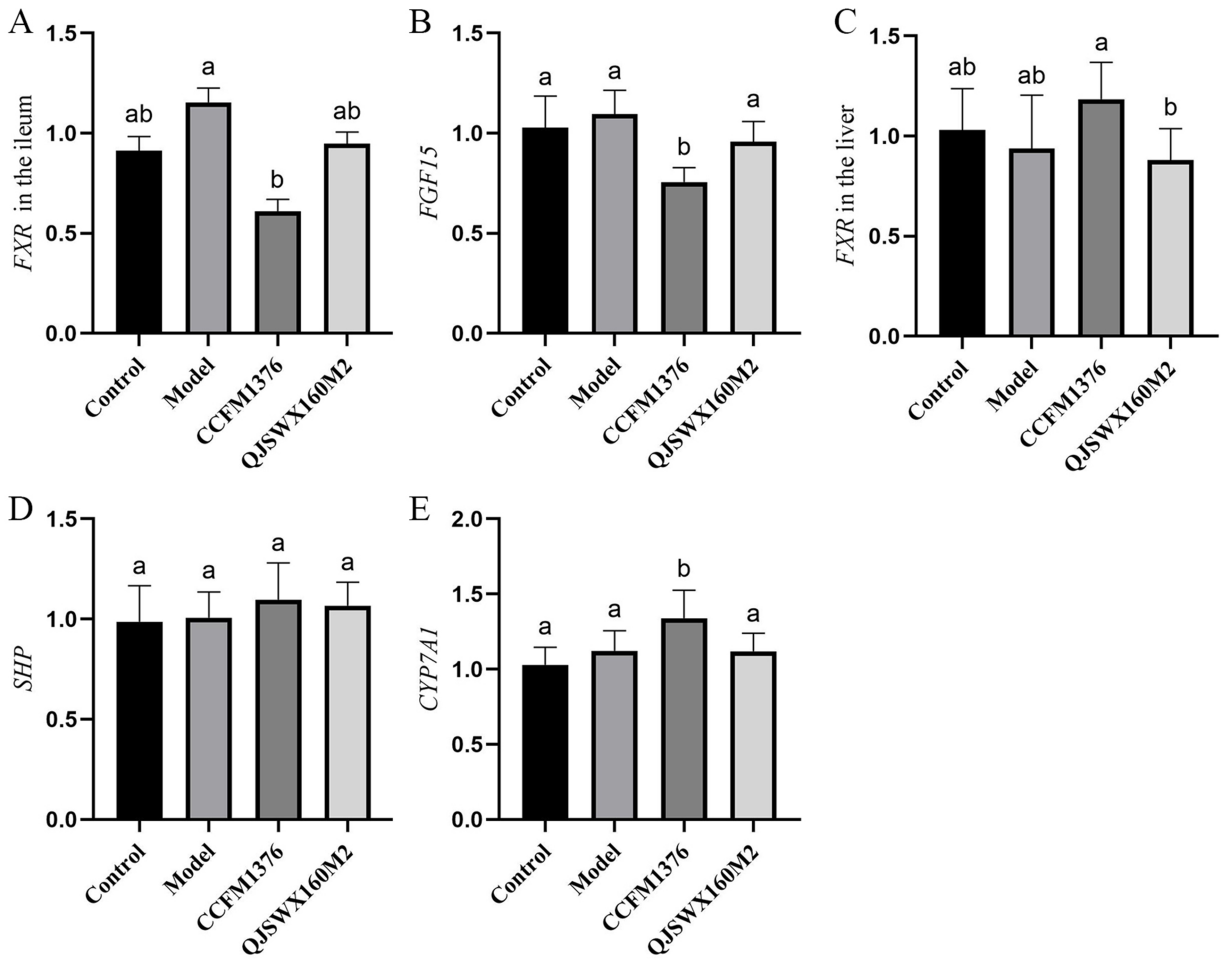
The effect of *L. johnsonii* CCFM1376 on the FXR signaling pathway in hypercholesterolemic mice. (A) FXR in the ileum; (B) FGF15; (C) FXR in the liver; (D) SHP; (E) CYP7A1. Different letters in each group indicated significant differences (*P* < 0.05). FXR: Farnesoid X receptor; FGF15: fibroblast growth factor 15; SHP: small heterodimer partner.

Small heterodimer partner (SHP) and fibroblast growth factor 15 (FGF15) are key signaling molecules in the liver FXR and intestinal FXR regulatory pathways, respectively. A study on the regulation of cholesterol metabolism by the active component of Pu-erh tea, theabrownin, has shown that the intestinal FXR-FGF15 and the liver FXR-SHP can dually regulate the expression of CYP7A1^[19]^. To investigate the impact of *L. johnsonii* CCFM1376 on the hepatic and intestinal FXR signalling mechanism in mice, the relative expression levels of the *FXR* and *SHP* in the liver, *FXR* and *FGF15* in the ileum, and the critical enzyme gene *CYP7A1* involved in liver bile acid production were measured. Compared to the model group, *L. johnsonii* CCFM1376 significantly downregulated the relative expression levels of *FXR* and *FGF15* in the mouse ileum, but did not significantly affect the relative expression levels of hepatic *FXR* and its key regulatory signaling gene *SHP* [*P* < 0.05, Figure 7A-D]. Concurrently, the CCFM1376 group significantly upregulated the relative expression level of *CYP7A1* [*P* <0.05, Figure 7E].

## DISCUSSION

In this study, we evaluated the alleviating effect of *L. johnsonii* CCFM1376, which possesses high BSH activity, on hypercholesterolemia in mice, and explored the impact of the bile acid metabolic pathway on this effect. The results indicated that *L. johnsonii* CCFM1376, with its high BSH activity, notably decreased serum TC and LDL-C levels in hypercholesterolemic mice, thus exerting a mitigating effect on hypercholesterolemia. Additionally, the bile acid composition in the liver and intestine of mice supplemented with *L. johnsonii* CCFM1376 was altered, and correspondingly, the expression profile of genes related to bile acid synthesis mediated by the FXR pathway was also modified.

A previous study has structurally identified a predictive circuit capable of discerning BSH preferences for glycine or taurine substrates through the detection of BSH diversity among intestinal Lactobacillaceae and other symbiotic bacteria^[13]^. Utilizing this circuit, a strain of *L. johnsonii* with three *BSH* genes was discovered, where two genes demonstrated a preference for glycine, while one gene exhibited a preference for taurine. *L. johnsonii* CCFM1376 has demonstrated a preference for glycine-conjugated bile acids. Under identical conditions, *L. johnsonii* BFE1061 exhibited a *BSH* activity of 0.2231 μmol·min^-1^·mL^-1^ toward glycodeoxycholic acid^[20]^. *L. johnsonii* BFE1061 has also been demonstrated to possess the ability to alleviate hypercholesterolemia in mice^[21]^.

In this study, mice fed a high-cholesterol diet exhibited altered blood lipid levels. Compared to the control group, the model group mice showed a notable rise in serum levels of TC and LDL-C. Supplementation with *L. johnsonii* CCFM1376 led to an improvement in blood lipid profiles, with significant reductions in serum TC and LDL-C levels, and a notable rise in HDL-C levels. Similarly, *L. johnsonii* BFE6154 significantly lowered the serum TC and LDL-C levels and markedly increased the serum HDL-C levels in hypercholesterolemic mice^[21]^. *L. johnsonii* N6.2 significantly reduced the serum TC levels in mice with diet-induced obesity^[22]^. The serum TG levels in mice on a high-cholesterol diet in this study did not show significant changes, which may be due to the type of diet fed, a finding that has also been confirmed in other studies utilizing the same dietary components^[14]^.

The liver is the primary site for cholesterol synthesis and metabolism in the human body. When the intake of cholesterol exceeds the body’s needs and the liver’s capacity to process it, cholesterol may accumulate in the liver. *L. johnsonii* CCFM1376 has been shown to reduce the levels of TC and LDL-C in the livers of mice with hypercholesterolemia. Similarly, *L. johnsonii* BFE6154 can also reduce the hepatic TC levels in hypercholesterolemic mice, but it does not have a considerable influence on the LDL-C levels in the liver^[21]^. *L. johnsonii* CCFM1376 can hydrolyze glycochenodeoxycholic acid (GCDCA) through its BSH activity, thereby inhibiting GCDCA-induced apoptosis in hepatocytes, and alleviating liver steatosis and bile acid dysregulation in parenterally nourished rats^[23]^.

Strain with *BSH* activity facilitates the production of various conjugated and unconjugated bile acids, as well as their metabolic products, secondary bile acids. These bile acids, under the influence of the hepatic-enteric circulation, alter the composition of bile acids within mice, and the distribution and variation of bile acids represent the material basis through which gut bacteria regulate cholesterol metabolism via BSH. Compared to the control group, mice on a high-cholesterol diet exhibited a significant increase in serum total bile acid concentration, indicating that a high-cholesterol diet leads to an imbalance in bile acid metabolism within the mice. A similar phenomenon is observed in mouse models where a high-fat diet results in obesity^[24]^. *L. johnsonii* CCFM1376 has a particularly significant impact on the content of bile acids in the terminal ileum and feces. The concentrations of several unconjugated bile acids, including β-MCA, CA, CDCA, UDCA, and HDCA, were notably increased in the terminal ileum of the *L. johnsonii* CCFM1376 group, consistent with the action of BSH. Similarly, after gavage administration of *Lactobacillus* with high BSH activity to normal mice, changes were observed in the mice’s bile acid composition, particularly in the ileum^[25]^. Prior to reaching the terminal ileum, approximately 95% of bile acids are reabsorbed, with unconjugated bile acids being less readily absorbed due to their higher hydrophobicity. An increased proportion of unconjugated bile acids in the feces of the *L. johnsonii* CCFM1376 group also confirms that strains with high BSH activity can elevate the content of unconjugated bile acids in the gut, accelerating the excretion of bile acids with feces. The BSH recombinant strain of *L. johnsonii* 334 modulated the fecal bile acid composition in mice on a high-cholesterol diet, with significant increases in the concentrations of unconjugated bile acid CA, β-MCA, DCA, and LCA^[26]^. Numerous studies have demonstrated that the intervention with Lactobacillus strains can regulate bile acid metabolism. For instance, *Lactobacillus plantarum* CCFM8661 can significantly increase bile acid synthesis in the liver and fecal bile acid excretion in mice^[27]^. In contrast, the supplementation with *Lactobacillus rhamnosus* GG can suppress de novo bile acid synthesis, reduce hepatic bile acid content, and increase bile acid excretion^[28]^.

Alterations in bile acid composition can influence the expression of key genes in the FXR pathway^[29]^. *L. johnsonii* CCFM1376 significantly downregulated the relative expression levels of FXR and FGF15 in the ileum of mice with hypercholesterolemia, but did not significantly affect the expression of FXR and SHP in the liver. Strains with BSH activity have shown varying effects on the ileum and liver; BSH recombinant strains downregulated the relative expression level of ileal FXR while upregulating hepatic FXR expression, ultimately demonstrating the ability to upregulate the relative expression of *CYP7A1*, thus promoting cholesterol metabolism. Joyce *et al.* found that the Lactobacillus BSH recombinant strain regulated plasma cholesterol levels in mice on a high-fat diet, with significant downregulation of hepatic FXR expression and significant upregulation of intestinal FXR expression^[9]^. Yao *et al.*, in their study on the effects of *BSH* gene knockout strains on lowering plasma cholesterol in mice on a high-fat diet, found significant downregulation of hepatic FXR expression with no significant change observed in intestinal FXR^[30]^. The action of gut microbiota BSH leads to an enhanced proportion of unconjugated bile acids in the gut, and the ileum’s relatively poor absorption of unconjugated bile acids, resulting in reduced reabsorption of bile acids in the ileum^[31]^. A decrease in intracellular bile acid levels in intestinal cells downregulates FXR transcriptional activity, leading to reduced levels of its downstream target gene FGF15 mRNA, thereby promoting the expression of CYP7A1. In the FXR regulatory pathway, Tβ-MCA and UDCA act as antagonists of the FXR receptor, while unconjugated bile acids such as CDCA are agonists^[32]^. Previous research has shown that compared to conjugated bile acids, unconjugated bile acids are less efficient in activating the FXR transcriptional mechanism in the ileum^[33]^. This also explains the significant increase in the concentration of the FXR agonist CDCA, the significant rise in the concentrations of the antagonists UDCA and Tβ-MCA, and the significant decrease in Tβ-MCA concentration in the ileum of the *L. johnsonii* CCFM1376 group, which collectively contribute to the suppression of ileal FXR. Unconjugated bile acid CDCA is less readily absorbed by intestinal cells and has a lower efficiency in activating FXR within these cells, allowing the antagonists Tβ-MCA and UDCA to predominate.

This study measured the levels of bile acids at various locations in the enterohepatic circulation of mice and detailed the direct link between the reduction of cholesterol and changes in bile acid composition. However, this study has its limitations, such as the lack of conclusive evidence to demonstrate the colonization of *L. johnsonii* CCFM1376 in the gut. Additionally, the fact that this study did not include more strains for comparison is also one of its limitations.

In summary, *L. johnsonii* CCFM1376 with high *BSH* activity can directly influence the composition of bile acids in the enterohepatic circulation of hypercholesterolemic conditions, promoting the conversion of conjugated bile acids to unconjugated bile acids. It also suppresses the expression of genes related to the FXR pathway in the mouse ileum, facilitating bile acid synthesis. Through the alteration of bile acid composition, *L. johnsonii* CCFM1376 exerts a mitigating effect on hypercholesterolemia in mice. This study provides a reference for the regulation of cholesterol metabolism by probiotic strategy.

## DECLARATIONS

### Availability of data and materials

All data are available in the main text. For other raw data, please contact the corresponding author.

### Copyright

© The Author(s) 2024.
